# A systematic approach for studying the signs and symptoms of fever in adult patients: the fever assessment tool (FAST)

**DOI:** 10.1186/s12955-017-0644-6

**Published:** 2017-04-27

**Authors:** Nancy J. Ames, John H. Powers, Alexandra Ranucci, Kyungsook Gartrell, Li Yang, Mark VanRaden, Nancy Kline Leidy, Gwenyth R. Wallen

**Affiliations:** 1 Clinical Center, National Institutes of Health, Bldg 10 Rm 2B-10, 10 Center Drive, Bethesda, MD 20892 USA; 2 Clinical Research Directorate/Clinical Monitoring Research Program, Leidos Biomedical Research, Inc., NCI Campus at Frederick, Frederick, Maryland 21702 USA; 3 National Library of Medicine/Lister Hill National Center for Biomedical Communications, and National Institutes of Health/Clinical Center Nursing Department, North Bethesda, Maryland USA; 4 National Institute of Allergy and Infectious Diseases, Bethesda, Maryland USA; 5 Evidera, Bethesda, Maryland USA

**Keywords:** Fever signs, Fever symptoms, Fever Assessment Tool (FAST)

## Abstract

**Background:**

Although body temperature is one of four key vital signs routinely monitored and treated in clinical practice, relatively little is known about the symptoms associated with febrile states. The purpose of this study was to assess the validity, reliability and feasibility of the Fever Assessment Tool (FAST) in an acute care research setting.

**Methods:**

Qualitative: To assess content validity and finalize the FAST instrument, 12 adults from an inpatient medical-surgical unit at the National Institutes of Health (NIH) Clinical Center participated in cognitive interviews within approximately 12 h of a febrile state (tympanic temperature ≥ 38° Celsius). Quantitative: To test reliability, validity and feasibility, 56 new adult inpatients completed the 21-item FAST.

**Results:**

The cognitive interviews clarified and validated the content of the final 21-item FAST. Fifty-six patients completed the FAST from two to 133 times during routine vital sign assessment, yielding 1,699 temperature time points. Thirty-four percent of the patients (N = 19) experienced fever at one or more time points, with a total of 125 febrile time points. Kuder-Richardson 20 (KR-20) reliability of the FAST was 0.70. Four nonspecific symptom categories, Tired or Run-Down (12), Sleepy (13), Weak or Lacking Energy (11), and Thirsty (9) were among the most frequently reported symptoms in all participants. Using Generalized Estimating Equations (GEE), the odds of reporting eight symptoms, Warm (4), Sweating (5), Thirsty (9), General Body Aches (10), Weak or Lacking Energy (11), Tired or Run Down (12) and Difficulty Breathing (17), were increased when patients had a fever (**Fever Now**), compared to the two other subgroups—patients who had a fever, but not at that particular time point, **(Fever Not Now)** and patients who never had a fever (**Fever Never**). Many, but not all, of the comparisons were significant in both groups.

**Conclusion:**

Results suggest the FAST is reliable, valid and easy to administer. In addition to symptoms usually associated with fever (e.g. feeling warm), symptoms such as Difficulty Breathing (17) were identified with fever. Further study in a larger, more diverse patient population is warranted.

**Trial Registration:**

Clinical Trials Number: NCT01287143 (January 2011)

## Background

Most individuals will experience symptoms of fever at some point in their lifetimes, whether the fever is from an immunological response that lasts for a few hours or from an infection spanning multiple days. Besides infection, the numerous causes of fever trigger a set of complex pathophysiological responses from central thermoregulators in the hypothalamus to peripheral nerves signaling vasodilation and the stimulation of sweat glands [1]. Healthcare providers rely on fever as a “vital sign” to alert them of these processes where they must intervene and order appropriate diagnostic tests [2, 3]. In some populations, such as neutropenic hosts, an abnormally high temperature is a sign that must be acted on quickly to prevent mortality [4, 5].

Despite the common prevalence of fever, there is little scientific evidence describing its signs and symptoms that would assist healthcare practitioners in recognizing, tracking and appropriately treating its symptomatic course. Descriptions in textbooks and journal articles, such as sweating, chills or shivering, are largely based on anecdotal, rather than reliable, empirical evidence and do not provide a comprehensive, evidence-based comparison of symptoms in febrile and afebrile states [6]. Further complicating the issue is the absence of a reference standard for body temperatures defining fever and the variance in devices used to record fever. Fever is an important and interesting clinical sign that deserves a clearer definition and an evidenced-based approach for investigating associated signs and symptoms.

The Fever Assessment Tool (FAST) was developed as a simple, standardized method for studying fever-associated signs and symptoms. Candidate items for this patient-reported questionnaire were based on results of semi-structured interviews with 28 medical-surgical inpatients conducted within approximately 12 h of a recorded fever (≥38 °C) and reported previously [7]. This research suggested patients experience a range of signs and symptoms during a febrile state including feeling cold, warm, and weak and informed the development of a draft instrument (FAST) of 21 signs and symptoms each with a yes/no response [7]. This manuscript describes the qualitative methods used to refine the instrument, assure content validity and perform quantitative analysis to assess reliability and validity of the FAST in a clinical setting prior to its use in a larger study.

## Methods

### Qualitative

The FAST was developed using a two-phase qualitative approach, with approval of National Cancer Institute’s intramural Institutional Review Board (Clinical Trials No. NCT01287143). Phase I provided the qualitative development work yielding the draft FAST. During Phase II, the draft FAST was administered to a new set of adult patients using the cognitive interviewing method. Cognitive interviews are one approach for understanding the utility and relevance of patient reported outcome (PRO) measures and documenting their content validity [8, 9]. This method provides investigators and clinicians with a tool for understanding individuals’ abilities to interpret PRO measures, the techniques they use for information retrieval from memory, their judgment formation on specific items and their editing of responses [9–11]. Inclusion criteria for the cognitive interviews involved an age of greater than or equal to 18, English language skills and interview occurrence within approximately 12 h of a fever. In this study, think-aloud and probing interview techniques were also used to evaluate the questionnaire for comprehension and clarity [12].

**Fig. 1:**
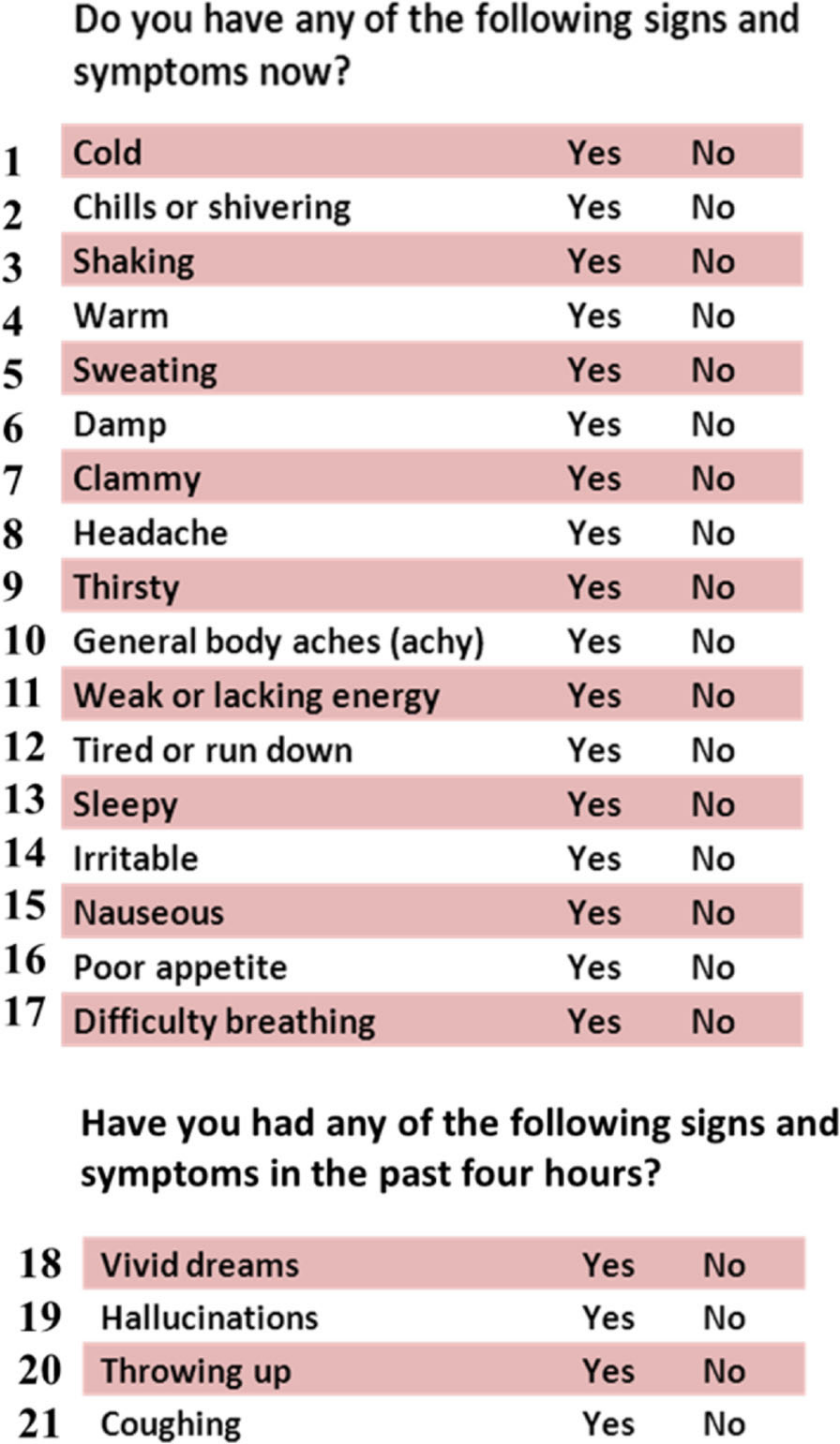
Fever Assessment Tool (FAST). Presents the fever assessment tool (FAST), developed from elicitation (*N* = 28) and cognitive interviews (*N* = 12) of patients who possessed fever within approximately 12 h of the interview. Sign and symptom numbers correspond to the text to assist in symptom identification

Twelve research participants completed the cognitive interviews from November 2011 to January 2012. The cognitive interviews served to: (1) assess whether the signs and symptoms selected for the FAST were understood as intended, (2) confirm that the measure possessed content validity and (3) identify data collection approaches or methods that might be used to enhance future data quality. The FAST included 21 items, each describing a sign or symptom associated with fever (Fig. 1). Seventeen of the items asked patients to positively or negatively indicate if they were presently experiencing the signs and symptoms (Fig. 1). The four remaining items, Vivid Dreams (18), Hallucinations (19), Throwing Up (20) and Coughing (21), asked about signs or symptoms within the past four hours (Fig. 1). Following the cognitive interviews and adjustments to the instrument, two additional patients participated in 15–20 min interviews to assess the ease of use of the FAST prior to instituting the pilot test in a larger number of patients.

### Quantitative

Reliability, validity and feasibility of the FAST were explored through an observational study performed on the medical-surgical oncology unit at the NIH Clinical Center from August 2013 through May 2014. A clinical nurse specialist informed the study team of admitted patients who were eligible; the study team subsequently screened those patients and, if appropriate, obtained written informed consent. Eligibility criteria included the following: greater than or equal to 18 years of age, admittance to the medical-surgical oncology unit, ability to speak English and willingness to answer yes or no questions about signs and symptoms at each vital sign intervention. All participants received care appropriate to their underlying condition and the investigational study under which they were admitted to the NIH Clinical Center.

Vital signs were recorded based on patients’ conditions and circumstances. Most vital sign orders stated the frequency as every four to 24 h. These orders were written by the attending physician based on patients’ medical conditions and main research protocol. The FAST was administered at each vital sign assessment unless the patient was medically unstable, unavailable, or refused. The FAST (Fig. 1) took fewer than 5 min to administer. Nurses or patient care technicians were instructed to read the assessment questions to the patient and ask him or her to indicate if each sign or symptom was present by giving a “yes” or “no” response.

Thermometers on this unit were all from the same manufacturer and used in the tympanic mode (Covidien/Kendall's Genius™ 2 infrared tympanic thermometer). In addition, a disposable oral digital thermometer was available (Medline Industries, Inc.), but rarely used. Other relevant information such as admitting diagnosis, antipyretic use and demographics were obtained from the Clinical Center’s clinical research information system, which serves as the electronic health record.

Prior to beginning data collection, the nurses and technicians attended education classes that reviewed the purpose of the study and taught the correct method to administer the FAST. In addition, updates and progress reports were provided to research staff. The principal investigator (N.A.) made rounds frequently over the 10 month data collection period, following up on consented patients, reviewing data quality, and providing study updates and progress to unit personnel.

### Statistical Analysis

The FAST was designed to study the prevalence of symptoms associated with the febrile state. The extent to which the items comprising the FAST are related to one another was estimated with data from the first observation (time point one) using the Kuder-Richardson-20 (KR-20) reliability coefficient for dichotomous response scales. KR-20 scores range from 0.0 to 1.00, with higher values indicating greater internal consistency among the items comprising an instrument [13]. Reliability estimates greater than 0.70 are acceptable, while values greater than 0.90 are ideal for instruments using an aggregate score for use in practice settings [14].

**Fig. 2:**
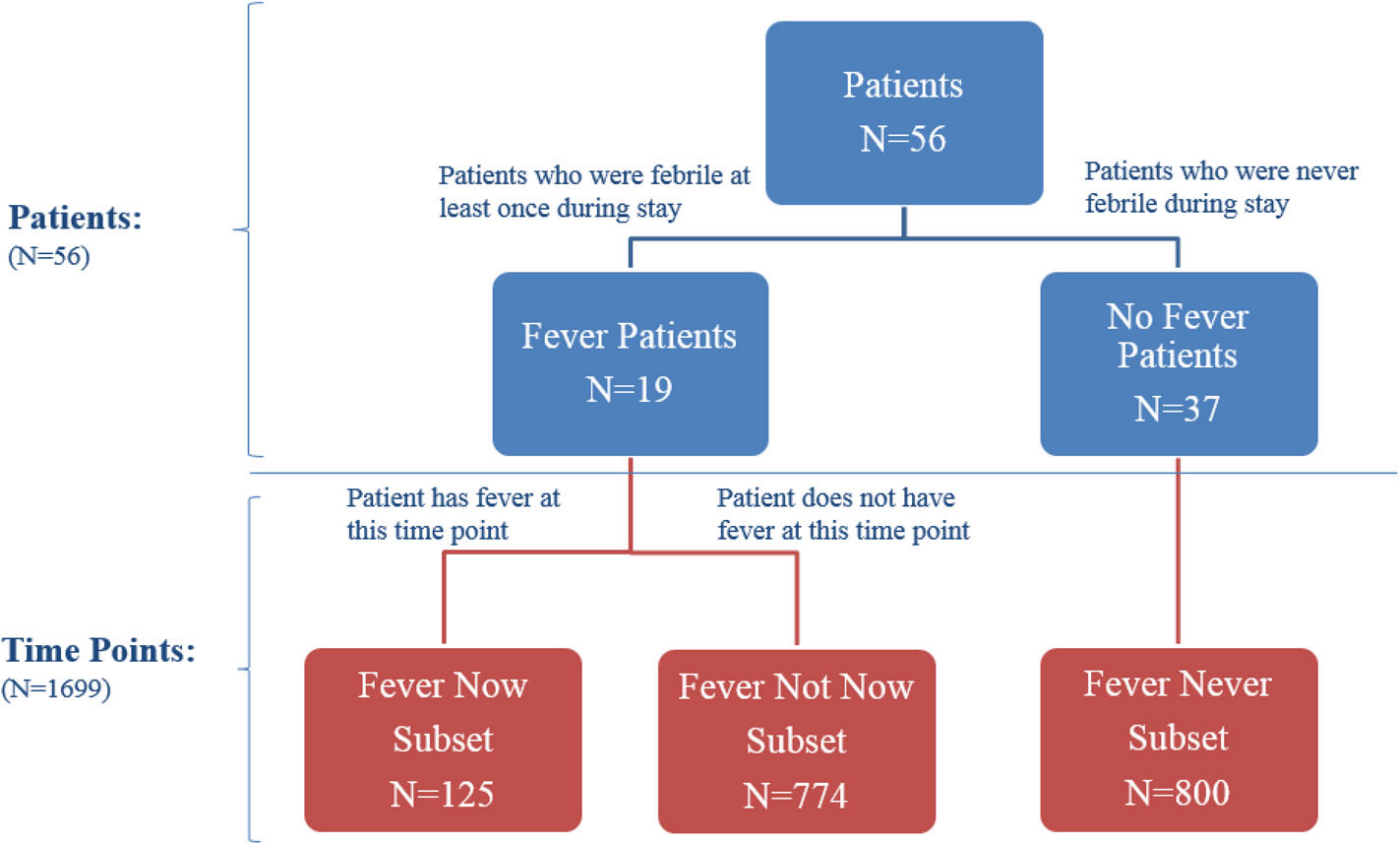
Schema of Study. This figure represents the schema of the study, distinguishing between patients and time point analysis. The **Fever Never** subset includes all time points of patients who never experienced fever on study. **The Fever** Now subset include only those time points when a fever was recorded. Patients who possessed at least one fever time point but did not record a fever at another time point were categorized into the **Fever Not Now** subset

Construct validity was assessed by examining the symptoms associated with febrile and afebrile states. Figure 2 shows the analytical schema for the study. Subjects are represented in the blue boxes while time points are in red. The time points are divided into three subsets: **Fever Not Now, Fever Now** and **Fever Never**. Subjects with a fever at one or more time points (Fever Patients) were divided into time points when fever was documented (**Fever Now**) and time points where fever was not present (**Fever Not Now)**. The remaining subjects represented those who ***never*** possessed fever during the study (No Fever Patients); therefore, measurements taken at all these time points were analyzed as the third subset (**Fever Never**). Construct validity would be supported if there was a difference in the symptoms reported across the three subsets, with particular interest in the **Fever Now** and **Fever Not Now** comparison.

Generalized Estimating Equations (GEE) were used to analyze the data. GEE is a type of estimation equation that models population level mean response for repeated measures with categorical and/or non-normal dependent variables related to logistic regression [15]. The results of this analysis with logit link function and first order autoregressive working correlation matrices were used to compare the odds of symptoms among the three subsets. Time was entered as a continuous variable in those models. A chi-square statistic based on the Wald test was obtained from the GEE analysis when contrasting any two of the three subsets. GEE with Poisson link function and unstructured working correlation matrix was used to evaluate symptom count between subsets. P values were considered significant if the value was less than 0.05.

Descriptive statistics were used to summarize the demographic characteristics of fever and non-fever cases. All analyses were performed using SAS (version 9.3, SAS, Cary, NC) or SPSS (version 21, IBM SPSS, Armonk, NY).

## Results

### Qualitative

Twelve interviews were conducted over a three month period to validate and clarify FAST language (Table 1). The majority of the 12 participants were white males and one-half of those interviewed were admitted for a planned surgery (Table 1). Nine patients received antipyretics within the previous 24 h period before the interviews. One patient received steroids and one patient was currently receiving chemotherapy within 24 h of the interview. Four patients had a diagnosis of metastatic melanoma. Cognitive interviews were recorded and duration ranged from a minimum of 5.5 min to a maximum of 39 min with a mean of 22 min. One interview was stopped after 9 min per the patient’s request because of pain.

**Table 1:** Demographic characteristics of patients who participated in cognitive interviews (*N* = 12)

Demographics	Cognitive Interview
Age mean (SD)	51.50 (13.18)
Minimum and maximum	25-74
Gender	*N* (%)
Male	7 (58.3%)
Female	5 (41.7%)
Race	
White	10 (83.3%)
Black/African-American	2 (16.7%)
Ethnicity	
Hispanic	1 (8.3%)
Surgery "Yes"	6 (50%)

Patient demographics of cognitive interview participants. A surgery “yes” response reports that the patient was admitted for surgery; during the admission, they consented to the interview

The words and phrases in the draft measure were clarified during these interviews. In the Phase I elicitation interviews, patients used the word “thirsty,” not “dehydrated.” During the cognitive interviews, “thirsty” was also characterized as more meaningful than “dehydrated.” Two terms, “clammy” and “damp,” seemed very close in meaning. However, patients felt that “clammy” was distinct and did not mean the same as “damp.” In almost all the interviews, patients agreed that they were different. Although the term “throwing up” is a sign, this language was preferred over “nauseous.” Finally, participants perceived “shaking” as different than “chills and shivering.” “Shaking” was considered a more intense sign than “chills and shivering.”

### Quantitative

#### Sample

Demographic characteristics of the quantitative sample (N = 56) are shown in Table 2. The majority were male (57%) and white (88%) (Table 2). There were four patients of Hispanic ethnicity (Table 2). Forty-five percent were admitted for surgery (Table 2). Surgical patients received pre-operative and post-operative care for cancer treatment, thoracic surgery and other surgical-driven protocols. The medical patients included those who received chemotherapy and/or other investigational drugs that required close monitoring (Table 2). Nineteen (34%) had a fever at some point during the study, with a total of 125 fever time points (**Fever Now**).

**Table 2:** Demographic characteristics of patients who provided FAST assessments (*N* = 56)

Demographics	Fever Patients	No Fever Patients	Total
	(*N* = 19)	(*N* = 37)	(*N* = 56)
Age Mean (SD)	54.21 (10.71)	54.70 (10.09)	54.5 (10.21)
Minimum and maximum	29-71	29-71	29-71
Gender	*N* (%)		
Male	10 (53%)	22 (59%)	32 (57%)
Female	9 (47%)	15 (41%)	24 (43%)
Race			
White	17 (89%)	32 (86%)	49 (88%)
Black/African-American	1 (5%)	3 (8%)	4 (7%)
Asian	0 (0%)	1 (3%)	1 (2%)
Other and Unknown	1 (5%)	1 (3%)	2 (4%)
Ethnicity			
Hispanic	0 (0%)	4 (11%)	4 (7%)
Other			
^a^Surgery "Yes"	14 (74%)	11 (30%)	25 (45%)
Chemo/Biotherapy "Yes"	6 (32%)	21 (57%)	27 (48%)
Steroids "Yes"	3 (16%)	8 (22%)	11 (20%)
^a^Antipyretics "Yes"	18 (95%)	15 (40%)	33 (59%)

^a^Statistically significant differences occur between Fever and No Fever Patients for both surgery (*p* = 0.004) and antipyretics (*p* < 0.001) using Fisher’s exact test.

Patient demographics in the FAST assessment (*N* = 56) categorized into two subsets: Fever Patients (*N* = 19) and No Fever Patients (N = 37). No other category is significant. A “Yes” answer to chemotherapy/biotherapy means that the patient received a chemotherapeutic/biotherapeutic agent during the admission where the symptoms were collected. Steroid and antipyretic use indicates that the patient had these drugs at some time during the admission where fever symptoms were collected

**Fig. 3:**
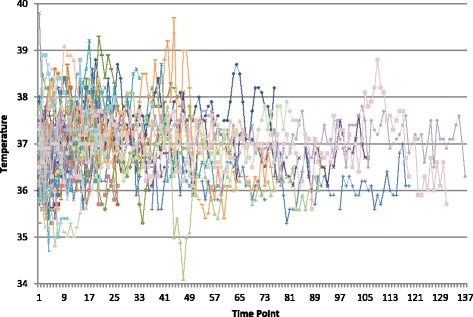
Scatter diagram of temperatures versus time points *N* = 1,699. Maximum recorded temperature was 39.8 °C while minimum temperature was 34.2 °C among 125 possible fever-qualifying temperatures. Eighty percent of the total readings qualified as fever (ranged 38.0 °C to 38.6 °C)﻿

Across the 56 patients there were 1,699 time points where vital signs were performed. The number of time points per patient ranged from 2 to 133. The mean number of time points with temperature recorded per patient was 30.3 (SD = 32.7). Figure 3 is a scatter diagram of temperatures versus time points (n = 1,699). The highest temperature recorded was 39.8 °C and the lowest was 34.1 °C (Fig. 3). The majority (80%) of the 125 temperatures that qualified as fever were between 38 °C and 38.6 °C.

Of the 1,699 time points, 282 (16.6%) were missing FAST data, yielding 1,417 time points with both temperature and FAST data. Five patients asked to stop answering the FAST questions due to worsening medical condition; data up to the point of discontinuation are included in the analyses.

#### Reliability

The Kuder-Richardson-20 (KR-20) reliability estimate was 0.695, based on data from 53 cases (three cases were excluded because of listwise deletion) and responses at the first time point. The first time point was used because as the study progressed, the sample size decreased, for example, by time point 6, the sample size was 40. This estimate included all 21 symptoms. Eight symptoms had no “yes” responses at the first time point in the study: Chills or Shivering (2), Shaking (3), Damp (6), Nauseous (15), Difficulty Breathing (17), Vivid Dreams (18), Hallucinations (19) and Throwing–up (20). After removing these eight from analysis, the alpha improved to 0.71.

#### Validity

Using the GEE model, average sign and symptom count per time point was significantly different between each pair of subsets (**Fever Now vs. Fever Not Now**, *p* =0.0092; **Fever Not Now vs. Fever Never**, *p* = 0.0026 and **Fever Now vs. Fever Never**, *p* = 0.0001). Comparing total symptom count by subset, **Fever Now** had at least two symptoms 59% of the time, while **Fever Not Now** and **Fever Never** reported at least two symptoms 49% and 31% of the time, respectively. The most common total symptom count in all three subsets was zero, or no symptoms. No symptoms were recorded in 43% of all time points, composing 29% of the **Fever Now**, 37% of **Fever Not Now** and 50% of **Fever Never** subsets.

**Fig. 4:**
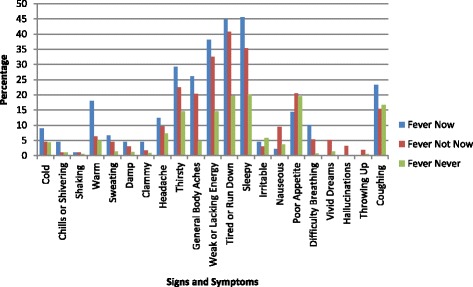
Frequencies of “yes” responses by patient subset across the 21 sign and symptom FAST assessment. “Yes” response frequencies (%) for each sign and symptom of the FAST by patient subset: **Fever Now, Fever Not Now** and **Fever Ne﻿ver**. The **Fever Now** category represents time points when a fever was recorded while the **Fever Never** group includes time points of patients who never experienced fever. Time points at which there was no fever **(F﻿ever Not Now)** included patients who had at least one febrile time point during the study duration

The FAST was designed to provide a symptom profile, showing the prevalence and signs and symptoms associated with fever. Table 3 outlines frequency of “yes” responses among the 21 FAST signs and symptoms and Fig. 4 displays these data in a bar chart. Across all time points and subsets, the following seven signs or symptoms were most commonly endorsed (>13% of the time): Thirsty (9), General Body Aches (10), Weak or Lacking Energy (11), Tired or Run Down (12), Sleepy (13), Poor Appetite (16) and Coughing (21). The following symptoms were observed to be more prevalent in the **Fever Now** subset relative to the **Fever Not Now** and **Fever Never** subsets: Cold (1), Chills or Shivering (2), Warm (4), Sweating (5), Damp (6), Clammy (7), Headache (8), Thirsty (9), General Body Aches (10), Weak or Lacking Energy (11), Tired or Run Down (12), Sleepy (13), Difficulty Breathing (17) and Coughing (21).

**Table 3:** Counts and frequencies of “yes” responses by patient subset across the 21 signs and symptoms assessed by the FAST

Signs and symptoms	Fever Now *N* = 90 N (%)	Fever Not Now *N* = 634 N (%)	Fever Never *N* = 693 N (%)	Totals *N* = 1417 N (%)
1. Cold	8 (9.0)	28 (4.5)	30 (4.4)	66 (4.7)
2. Chills or shivering	4 (4.5)	7 (1.1)	7 (1.0)	18 (1.3)
3. Shaking	1 (1.1)	7 (1.1)	4 (0.6)	12 (0.9)
4. Warm	16 (18.0)	40 (6.3)	34 (5.0)	90 (6.4)
5. Sweating	6 (6.7)	28 (4.4)	9 (1.3)	43 (3.0)
6. Damp	4 (4.4)	19 (3.0)	8 (1.2)	31 (2.2)
7. Clammy	4 (4.4)	11 (1.8)	6 (0.9)	21 (1.5)
8. Headache	11 (12.4)	61 (9.7)	51 (7.4)	123 (8.7)
9. Thirsty	26 (29.2)	142 (22.5)	100 (14.6)	268 (19.0)
10. General body aches	23 (26.1)	128 (20.4)	33 (4.8)	184 (13.1)
11. Weak or lacking energy	34 (38.2)	205 (32.5)	100 (14.6)	339 (24.1)
12. Tired or run down	40 (44.9)	258 (40.8)	134 (19.6)	432 (30.8)
13. Sleepy	41 (45.6)	223 (35.4)	138 (20.0)	402 (28.6)
14. Irritable	4 (4.5)	19 (3.0)	40 (5.8)	63 (4.5)
15. Nauseous	2 (2.3)	60 (9.5)	25 (3.6)	87 (6.2)
16. Poor Appetite	13 (14.4)	129 (20.4)	134 (19.5)	276 (19.6)
17. Difficulty Breathing	9 (10.1)	33 (5.2)	5 (0.7)	47 (3.4)
18. Vivid dreams	0 (0)	32 (5.1)	9 (1.3)	41 (3.0)
19. Hallucinations	0 (0)	20 (3.2)	1 (0.2)	21 (1.5)
20. Throwing up	0 (0)	11 (1.8)	4 (0.6)	15 (1.0)
21. Coughing	20 (23.3)	96 (15.4)	114 (16.7)	230 (16.6)

“Yes” response counts (n) and frequencies (%) for each sign and symptom of the FAST by patient subset: **Fever Now** (*N* = 90), **Fever Not Now** (*N* = 634) and **Fever Never** (*N* = 693) reported by time point. The number of “yes” responses are divided by the number of times each symptom was answered by participants within the column. The final totals column includes 1,417 time points with temperature and FAST assessment. Two hundred and eighty-two temperatures were obtained without a FAST completed. The total sample size in each subgroup is the total time points. However, sample sizes for each symptom slightly differ because of missing data

GEE were performed to statistically analyze these symptoms in the **Fever Now** and **Fever Not Now** subsets, using the **Fever Never** subset as the default comparison group (values set at zero). Table 4 displays GEE results for eight symptoms that showed significant differences. The odds ratio is also supplied for the comparisons (Table 4). The following eight symptoms were significantly more likely to be included in the **Fever Now** subset when comparing **Fever Now** with the reference subset **Fever Never**: Warm (4), Sweating (5), Thirsty (9), General Body Aches (10), Weak or Lacking Energy (11), Tired or Run Down (12), Sleepy (13) and Difficulty Breathing (17) (Table 4). Odds ratios ranged from 3 to 19, with the odds of Difficulty Breathing (17) 19 times as likely (95% CI 4.26 to 46.99) in the **Fever Now** subset as compared to F**ever Never** (Table 4). Six symptoms showed significance when the **Fever Not Now** and **Fever Never** were compared: Sweating (5), General Body Aches (10), Weak or Lacking Energy (11), Tired or Run Down (12), Sleepy (13) and Difficulty Breathing (17), all of which were also significant for **Fever Now** versus **Fever Never.**

**Table 4:** Odds ratio of observing eight statistically significant signs and symptoms of fever by subset

Signs and symptoms	β (SE)	Odds ratio (95% Confidence Interval)	*p* value
4. Warm			
Fever Now	1.57 (0.52)	4.81 (1.73-13.33)	0.0025
Fever Not Now	0.24 (0.53)	1.27 (0.450-3.63)	0.6491
5. Sweating			
Fever Now	1.88 (0.56)	6.55 (2.20-19.69)	0.0007
Fever Not Now	1.28 (0.55)	3.60 (1.22-10.49)	0.0198
9. Thirsty			
Fever Now	1.14 (0.48)	3.13 (1.22-8.00)	0.0166
Fever Not Now	0.51 (0.40)	1.67 (0.76-3.63)	0.1990
10. General body aches			
Fever Now	2.32 (0.53)	10.18 (3.53-29.37)	<.0001
Fever Not Now	1.62 (0.38)	5.05 (2.41-10.70)	<.0001
11. Weak/lacking energy			
Fever Now	1.71 (0.60)	5.53 (1.70 -18.17)	0.0045
Fever Not Now	1.04 (0.46)	2.83 (1.15-7.03)	0.0238
12. Tired/run down			
Fever Now	1.48 (0.45)	4.39 (1.80-10.59)	0.0010
Fever Not Now	1.05 (0.39)	2.86 (1.34-6.11)	0.0067
13. Sleepy			
Fever Now	1.50 (0.45)	4.48 (1.84-11.02)	0.0009
Fever Not Now	0.75 (0.36)	2.12 (1.05-4.26)	0.0346
17. Difficulty Breathing			
Fever Now	2.95 (0.76)	19.11 (4.26-46.99)	0.0001
Fever Not Now	2.01 (0.76)	7.46 (1.70-32.79)	0.0079

Odds ratio and GEE (β) estimate with accompanying standard error (SE) for eight statistically significant symptoms of fever reported for either **Fever Now** and/or **Fever Not Now** subsets, where the **Fever Never** subset represents the default comparison group (values set at zero). The **Fever Never** subset included all time points in patients who never experienced fever. The **Fever Now** are those time points when a fever was recorded. Patients who had at least one time point of fever, but did not possess fever at the time point were categorized as the **Fever Not Now** subset

The GEE contrast analyses showed significant differences in “yes” responses between the contrasts **Fever Now** and **Fever Not Now** for 5 symptoms, Chills or Shivering (2) (*χ* ^2^ = 7.42; *p* = 0.006), Warm (4) (*χ* ^2^ = 8.89; *p* = 0.003), General Body Aches (10) (*χ* ^2^ = 5.88; *p* = 0.015), Sleepy (13) (*χ* ^2^ = 4.27; *p* = 0.039) and Difficulty Breathing (17) (*χ* ^2^ = 4.10; *p* = 0.043) (Table 5).

**Table 5:** Statistically significant signs and symptoms of fever between Fever Now and Fever Not Now subsets

Signs and Symptoms	*χ* ^2^	*p*
2. Chills or Shivering		
Category	7.42	0.024
Fever Now vs. Fever Not Now	7.42	0.006
4. Warm		
Category	12.98	0.0015
Fever Now vs. Fever Not Now	8.89	0.003
10. General Body Aches		
Category	19.66	<0.0001
Fever Now vs. Fever Not Now	5.88	0.015
13. Sleepy		
Category	10.96	0.004
Fever Now vs. Fever Not Now	4.27	0.039
17. Difficulty Breathing		
Category	15.49	0.0004
Fever Now vs. Fever Not Now	4.10	0.043

Five statistically significant signs and symptoms across **Fever Now** and **Fever Not Now.** The category is overall comparison among all three groups. It is included (Wald statistics *χ* ^2^ and p values) because this value must be significant before examining the contrasts **Fever Now** and **Fever Not Now** in the GEE contrast analyses

## Discussion

The FAST was developed to address the need for a simple, standardized approach for studying fever-associated signs and symptoms. The 21 “yes/no” signs and symptoms comprising the instrument reflect the signs and symptoms experienced by patients during febrile states, employing terminology used by the patients themselves [7]. This manuscript describes the methods used to further assess the FAST’s content validity and reliability of the instrument. Overall, the FAST is easy for respondents to comprehend and requires fewer than 5 min to administer.

A finding supporting the validity of the FAST is the significant difference found in the average sign and symptom count per time point between each subset pair **(Fever Now** vs. **Fever Not Now**, *p* =0.0092; **Fever Not Now** vs. **Fever Never**, *p* = 0.0026 and **Fever Now** vs. **Fever Never,** *p* = 0.0001). Although there is a significant difference between **Fever Not Now** vs. **Fever Never**, this could indicate a difference in symptom development over time. This study did not control for temperature timing before or after fever; therefore, the **Fever Not Now** group included patients that had a prior fever at some point throughout study duration. This timing was not controlled and varied within patients. This difference between **Fever Not Now** vs. **Fever Never** needs further testing in a larger sample. Although some of these signs and symptoms are “non-specific” and may occur in many diseases states, these data show that the identified signs and symptoms are specifically associated with the fever state. The extent to which more symptomatic fevers are associated with greater patient distress or are predictive of adverse health outcomes are empirical questions for future study.

In general, most clinicians equate chills, coldness, warmth, and shivering with fever given their assumed specificity for diagnosing the febrile state. Results of the GEE comparison between **Fever Now** and **Fever Not Now** subsets suggest additional symptoms may also characterize fever, including General Body Aches (10), Feeling Sleepy (13) and Difficulty Breathing (17) (Table 4).

Qualitative methods addressed the content validity of the measure. The KR-20 value of 0.695 suggests the FAST total score reliably indicates the relatedness of these signs and symptoms [16] while the profile analyses offer insight into patient experiences during febrile and afebrile states.

Two respiratory symptoms, Coughing (21) and Difficulty Breathing (17), were included in the FAST (Fig. 1). Difficulty Breathing (17) was significantly different between both contrasts in the GEE analysis (Table 4). The odds of possessing a fever at the same time as answering “yes” to Difficulty Breathing (17) was estimated as 19 times higher compared to the rate differential seen in the **Fever Never** group (Table 4). Although Difficulty Breathing (17) was not as common as Coughing (21), it was reported at a total of 47 time points (Table 3). When patients were asked if they experienced coughing in the past four hours, 230 patients responded “yes” (Table 3). Coughing has not been previously associated with fever except, of course, in patients that have respiratory infections. Although it is possible that some of the patients in this study had underlying respiratory symptoms, the majority were not admitted for a primary respiratory illness. Post-operative patients are asked to “cough and deep breathe” as a routine part of their post-operative care. In this study, 45% were admitted for surgery (Table 2). Across all subsets, coughing was reported as a “yes” response at approximately 16% (230/1389) of the time points (Table 3). In **Fever Now** patients, 23% (20/86) of the time points reported coughing within the past four hours (Table 3). Coughing did not have a statistically significant difference by fever subset, although patients answered “yes” frequently.

The results of this study suggest the FAST detected symptomatic differences between febrile and afebrile states and that signs and symptoms of fever may go beyond warmth and chills. Further, a substantial proportion of patients may not experience symptoms with fever. This calls into question both the common practice of administering antipyretics to all febrile patients to purportedly improve symptoms and using fever as a surrogate endpoint for patient symptoms in clinical trials. The extent to which symptoms vary by fever magnitude or over the peri-febrile period, i.e., as temperatures rise and fall, and the extent to which asymptomatic patients may represent a specific phenotype or experience different health outcomes should be addressed in a future study with a larger and more diverse sample. In the absence of empirical evidence supporting the appropriate treatment of fever, clinicians often order antipyretics in acute care settings to treat the fever and relieve symptoms [17]. Even experts cannot agree if the symptoms of fever are beneficial in improving outcomes in the underlying diseases [18]. The relationship between fever, symptom count or type, antipyretic treatment, and health outcomes should also be examined through future studies.

Three items were never endorsed in the **Fever Now** subset: Vivid Dreams (18), Hallucinations (19) and Throwing Up (20), while Shaking (3) was only endorsed once, suggesting they are possible candidates for deletion (Table 3). However, given the limitations of this sample and the ease with which the FAST can be administered in its current form, these items will be retained for the next study.

The odds of reporting the following eight symptoms, Warm (4), Sweating (5), Thirsty (9), General Body Aches (10), Weak or Lacking Energy (11), Tired or Run Down (12), Sleepy (13) and Difficulty Breathing (17), were significantly increased in **Fever Now** patients compared to the **Fever Never** subset (Table 4). The comparison of the **Fever Now** and **Fever Never** subsets is the strongest as it compares time points where patients possessed fever to time points where patients did not have a fever at that time point or any time in their stay.

Using the GEE and examining contrasts, the comparison between **Fever Now** and **Fever Not Now** is also interesting as both of these subsets are composed of the same patients (Table 5). Nevertheless, despite the small sample of 19 patients with fever in the study (Fig. 2), five FAST symptoms: Chills or Shivering (2), Warm (4), General Body Aches (10), Sleepy (13), and Difficulty Breathing (17), were significantly more common at febrile time points (Table 5). The fact that only five significant symptoms of fever were found could be due not only to the small sample size, but also to other confounding factors, including the administration of antipyretics. Typically, depending on the severity of a patient’s symptoms and his or her temperature, the nurse would administer an antipyretic and re-check the temperature in an hour or two hours. All but one of the patients who reported a fever in this study received an antipyretic at some time. Nevertheless, treating fevers with antipyretics was a common practice. This intrapatient result does suggest that these specific symptoms were not simply the result of hospitalization, but rather, changed depending on a patient’s febrile state. A larger sample size and more standardized vital sign assessments are still necessary to further support this hypothesis.

### Limitations

The study team anticipated some missing data because of surgical procedures, diagnostic examinations and other interruptions when the patient would not be present on the unit. In addition to such events, there were many instances where vital signs were obtained and the nurse or the technician forgot or the patient refused the FAST. In some FAST checklists, items were missed or illegible. To minimize missing data, the study team trained the nursing staff prior to the initiation of data collection, made daily rounds on the unit, reminded staff of the patients on study and performed numerous inservices and follow-ups with the nurses during the 10-month data collection period. Despite these steps, approximately 17% of the time points with vital sign and/or temperature data did not have a corresponding completed FAST. In addition, there were times when the nurse did the FAST, but there was no recorded temperature.

A second limitation of the study was the broad use of antipyretic treatment, which may have reduced the frequency of fever, symptom reporting, and/or their co-occurrence. Many of the patients with a fever episode received an antipyretic at some time during the observation period, although antipyretic use could only be analyzed by patient, not by time point. Nevertheless, intrapatient analyses showed that Chills or Shivering (2), Warm (4), General Body Aches (10), Sleepy (13), and Difficulty Breathing (17) varied by febrile state, suggesting these symptoms could be attributable to fever regardless of antipyretic treatment (Table 5). Further study is needed in a larger sample and more diverse population to test this hypothesis.

Third, we did not assess the health literacy of the study participants when developing the FAST. However, cognitive interviews were conducted thoroughly for validation and clarification of medical language in the FAST. The cognitive interviews were sufficient to assure patient understanding and his or her ability to interpret medical language.

Fourth, data were collected on a medical-surgical oncology unit of a clinical research hospital where the patient population was presumably different from a community hospital or even an academic medical center’s medical-surgical oncology unit. The lack of diversity in the sample population presents another limitation.

Finally, the generalizability should be cautiously viewed due to the small sample size. However, one of the purposes of this pilot study was to assess the measure’s feasibility in a clinical setting, so no primary hypothesis was chosen and hence sample size was not formally calculated. Study data showing a significant association between fever and its signs and symptoms were assessed with a sufficient number of time points where a temperature was identified. Thirty-four percent of the 56 patients had a fever at some point in the study. There were 125 time points collected that were categorized as fever. Thus, this pilot study was the first step in building a reliable tool to assess signs and symptoms of fever. The sample size of 56 was sufficient to establish preliminary reliability of the measure. Future work will involve using the FAST in different patient populations and settings.

## Conclusion

Results of this study suggest the FAST is simple for patients to understand and easy for clinicians or technicians to administer. Overall, this study examined if there was a difference in symptoms experienced by febrile and afebrile patients. Preliminary evidence demonstrates a significant difference in the odds of encountering specific symptoms in the febrile state, with greater variety of symptoms associated with fever than usually reported in the literature. The data also show a substantial proportion of patients with seemingly asymptomatic fever. Further use of the FAST to track symptomatic change over time in febrile and afebrile states in a larger, more diverse sample is, therefore, warranted.

## Abbreviations

FAST: Fever Assessment Tool
GEE: Generalized Estimating Equations
KR-20: Kuder-Richardson 20
NIH: National Institutes of Health
PRO: Patient Reported Outcome
